# PTH after Thyroidectomy as a Predictor of Post-Operative Hypocalcemia

**DOI:** 10.3390/diagnostics11091733

**Published:** 2021-09-21

**Authors:** Alessio Metere, Andrea Biancucci, Andrea Natili, Gianfrancesco Intini, Claire E. Graves

**Affiliations:** 1 General Surgery Department, Ospedale dei Castelli (O.D.C.), Via Nettunense Km 11,5, 00040 Ariccia, Italy; andrea.natili85@gmail.com; 2 Surgical Sciences Department, “Sapienza” University of Rome, Viale Regina Elena 261, 00161 Rome, Italy; andreabiancucci@gmail.com; 3 General Surgery Department, Anzio Hospital, Via Cupa dei Marmi snc, 00042 Anzio, Italy; gianfrancesco.intini@gmail.com; 4 Department of Surgery, University of California, San Francisco 1600 Divisadero St. 7th Floor, San Francisco, CA 94115, USA; claire.e.graves@gmail.com

**Keywords:** thyroid surgery, PTH levels, hypocalcemia post-thyroidectomy

## Abstract

Post-thyroidectomy hypocalcemia is a frequent complication with significant morbidity, and has been shown to increase hospital stay and readmission rates. The evaluation of serum parathyroid hormone (PTH) levels after thyroidectomy represents a reliable method to predict post-thyroidectomy hypocalcemia, but it remains infrequently used. This retrospective study investigates serum PTH values 3 h after thyroidectomy as a predictor of hypocalcemia. In this study, we enrolled 141 patients aged between 27 and 71 years eligible for total thyroidectomy who presented with multinodular goiter, suspicious nodule on cytological examination, Graves’ disease, or toxic multinodular goiter. Three hours after total thyroidectomy, 53 patients (37.6%) showed a reduction in serum PTH. Of these patients 75.5% developed hypocalcemia by 24 h after surgery and 100% were hypocalcemic after 48 h (*p* < 0.001). There was no significant difference attributable to the different thyroid diseases, nor to the age of the patients. PTH at 3 h after total thyroidectomy accurately predicts post-operative hypocalcemia. The early detection of patients at risk of developing post-operative hypocalcemia allows for prompt supplementation of calcium and Vitamin D in order to prevent symptoms and allows for a safe and timely discharge.

## 1. Introduction

**Figure 1:**
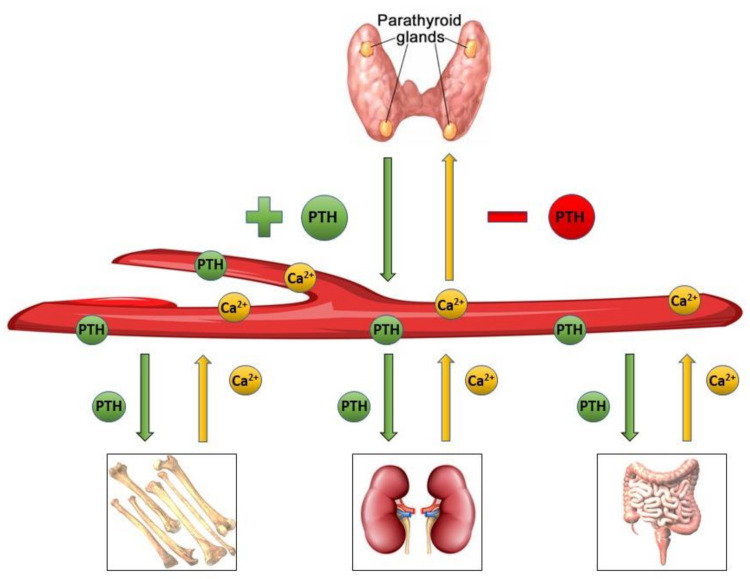
Serum PTH levels are tightly regulated by a negative feedback loop. PTH is secreted by the parathyroid glands as serum calcium levels drop, inducing the bones to release more ionic calcium and stimulating the kidney and intestine to reabsorb calcium. The release of PTH is reduced as serum calcium levels increase.

Hypocalcemia following thyroidectomy is a frequent complication with significant morbidity and has been shown to increase hospital stay and readmission rates [1]. Post-thyroidectomy hypocalcemia is due to hypoparathyroidism caused by devascularization or inadvertent resection of the parathyroid glands [2]. Depending on the type of damage and the number of glands involved, hypocalcemia may be transient or permanent. Preservation of the parathyroid glands during thyroid resection requires accuracy, experience, and expert knowledge of the neck anatomy [3]. The parathyroid glands vary in position (inferior parathyroid glands being more widely distributed than the superior glands), number, size, shape, and color [4]. If a parathyroid gland is clearly ischemic or hemorrhagic, auto-transplantation should be considered after confirmatory histological examination. Surgical samples should always be investigated for parathyroid tissue on the specimen, as invertedly resected glands can be auto-transplantated at the end of the operation to prevent long-term hypoparathyroidism [5,6]. Methods to prevent post-thyroidectomy hypocalcemia remain debated among surgeons, and multiple protocols have been proposed. Some surgeons prefer to administer calcium and/or vitamin D only in patients with symptoms of hypocalcemia or corrected serum calcium less than 8.5 mg/dL [7]. Others prefer prophylactic calcium supplementation to prevent postoperative hypocalcemia without routine laboratory tests [8]. Finally, some surgeons use serum parathyroid hormone (PTH) values to identify patients that require calcium and/or vitamin D supplementation [9,10]. PTH is an 84-amino acid protein secreted by the parathyroid glands in order to maintain calcium homeostasis. PTH secretion is increased in response to low serum calcium levels due to calcium receptors, present on the parathyrocytes, which regulate PTH secretion through a direct feedback mechanism based on the extracellular calcium ion concentration. In brief, the main role of the PTH is to induce the bones to release more ionic calcium and stimulate the kidney and intestines to reabsorb calcium (Figure 1). A decrease in PTH levels may induce a clinically detectable hypocalcemia delayed for up to 48 h after the hypoparathyroidism. The development of hypocalcemia depends on several factors, including the catabolism of PTH (some PTH particles continue to have biological activity), vitamin D levels, and the presence of bone mineral disorders [11,12]. These factors are important for surgeons to take into account in order to prevent the development of post-thyroidectomy hypocalcemia. As previously described, the routine administration of calcium and/or vitamin D in the post-operative period (regardless of serum calcium levels) meaningfully reduces the incidence of symptomatic hypocalcemia. At the same time, the routine use of calcium and/or vitamin D supplementation could be considered overtreatment, as not all patients need calcium and/or vitamin D after thyroidectomy. The assessment of early post-operative serum PTH helps predict the subsequent development of hypocalcemia. This allows for selective and appropriate early supplementation to reduce the risk of symptoms or complications, while facilitating early postoperative discharge. This study aims to evaluate the capacity of early serum PTH to predict the development of post-surgical hypocalcemia after thyroidectomy.

## 2. Materials and Methods

### 2.1. Recruitment of Patients

Study patients were recruited prospectively at the Department of Surgical Sciences of the Umberto I Hospital in Rome. We enrolled 141 patients aged between 27 and 71 years who underwent total thyroidectomy for multinodular goiter (MNG), a suspicious nodule (TC) on cytological examination after fine needle aspiration (according to the classification of the Italian Society of Pathology and Diagnostic Cytopathology [13]), Graves’ disease, or toxic (multi)nodular goiter (TG) (Table 1). We enrolled a similar number of patients for each category to statistically verify whether thyroid pathology may influence serum PTH levels. Histological examination showed that all patients undergoing total thyroidectomy for suspicious nodules were confirmed to have differentiated thyroid carcinoma (papillary or follicular). Patients were excluded from the study if they had debilitating diseases (advanced stage of diabetes, immunological diseases, or hematological disorders) or lack of informed consent and/or authorization for the processing of personal data. All procedures involving human participants were in accordance with the 1964 Helsinki declaration and its later amendments, or comparable ethical standards, and with the ethical standards of the ethics committee of the Umberto I Hospital of Rome that approved the study (Id number: 4240). All patients provided written informed consent for the use of clinical specimens for medical research.

### 2.2. Surgical Treatment

Surgical treatment was planned considering the cytological diagnosis, lymph node involvement, and the number and the size of the suspicious nodule(s) (based on US of the neck), age of patients, and history of prior head and neck irradiation. Though thyroid lobectomy alone may be sufficient treatment for small, low risk, unifocal, intrathyroidal papillary carcinomas in the absence of prior head and neck irradiation or radiologically or clinically involved cervical nodal metastases, as suggested by ATA 2015 [14], we included in this study only patients undergoing total thyroidectomy. Moreover, all patients requiring neck dissection were excluded from this study in order to obtain a more homogeneous population. The thyroidectomy was performed by only two surgeons experienced in thyroid surgery to reduce operator variability.

### 2.3. PTH and Calcium Analyses and Hypocalcemia/Hypoparathyroidism Management

Serum PTH levels were obtained before the induction of anesthesia and again 3 h after thyroidectomy. All tests were performed in the same laboratory with the same technique, and they were usually ready within 1 h after collection. Serum calcium levels were detected before surgery and at 24 and 48 h after the thyroidectomy. The normal range of serum PTH levels was considered between 15 and 65 pg/mL, while the normal range of serum calcium levels was between 8.5 and 10.5 mg/dl. All patients had serum PTH and calcium levels within the normal range before surgery. After surgery, patients whose PTH was <15 pg/mL and/or serum calcium was <8.5 mg/dL were considered for postoperative oral calcium and vitamin D supplementation. A regimen of 400–1200 mg per day of elemental calcium (1–3 g of calcium carbonate; i.e., 2–6 TUMS per day) or the equivalent in calcium citrate (2000–6000 mg per day) administered orally in divided doses was usually sufficient. Vitamin D (calcitriol, typically 0.25–0.5 mcg twice daily), was added to their regimen in the case of a decrease in the serum calcium levels on sequential measurements or if the values remained <7 mg/dL [15]. As chronic Mg deficiencies could influence PTH secretion, we also evaluated the serum magnesium concentration in all patients before and after thyroidectomy.

### 2.4. Laboratory Analyses

The routine assay used for serum PTH analyses was an immunometric “sandwich-type” to detect intact PTH (IMMULITE 2000 XPi Immunoassay System, Siemens). In brief, these 2nd generation assays are based on two different monoclonal antibodies, linking the amino-terminal and the carboxyl terminal site of PTH, respectively, to detect intact PTH. These assays are typically the most common type used in hospital laboratories. Serum calcium levels were determined by colorimetric assay.

### 2.5. Statistical Analyses

Data were analyzed using GraphPad version 5.0 software. Differences between the samples before and after thyroidectomy were calculated with the unpaired Student’s *t*-test, while the differences between the disease groups were calculated with the one-way ANOVA test. The linear regression was used to assess the relation between PTH and calcium levels. Significance was set at 95% confidence interval (*p* < 0.05).

## 3. Results

### 3.1. Study Population

**Figure 2:**
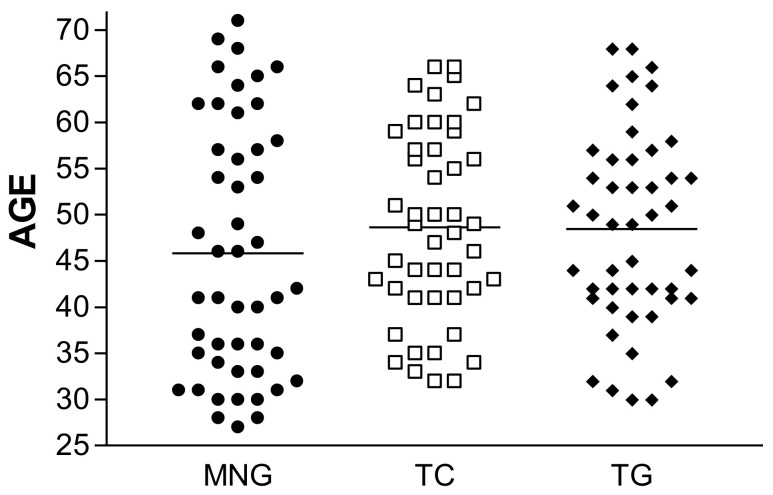
Age distribution and mean age for each category of disease. There was no significant difference between the groups (*p* = 0.42). MNG: multinodular goiter, TC: thyroid cancer, TG: Graves’ disease or toxic (multi)nodular goiter.

Table 1 describes characteristics of the study population. Patients were predominately female (75.2%), and the mean age was 48.73 ± 13.44 years. As previously described, to prevent variation due to the type of surgery (total thyroidectomy, thyroid lobectomy or total thyroidectomy plus total neck dissection), only patients undergoing total thyroidectomy were enrolled. A comparable number of patients with each type of thyroid disease (multinodular goiter, thyroid cancer, and Graves’ disease or toxic (multi)nodular goiter) were enrolled. The MNG group was composed of 48 patients aged between 27–71 years old (mean 45.81 ± 13.61), the TC group was composed of 46 patients aged between 32–66 years old (mean 48.65 ± 10.23) and the TG group of 47 patients 30–68 years old (mean 48.43 ± 10.62). Each category was sex and age matched. Statistical analysis demonstrates that for each group of disease the patients were correctly age and sex matched, and no difference was detected between the groups (*p* = 0.42, Figure 2).

### 3.2. Analyses of Serum Levels of PTH and Calcium

The serum PTH and calcium levels detected before surgery were homogenous and within the normal range, and no statistical difference was observed between the groups before thyroidectomy (*p* > 0.05). Three hours after total thyroidectomy, 53 patients (37.6%) showed a reduction in serum PTH characterized by levels below the normal range. Forty patients (28.4% of the overall cohort and 75.5% (40/53) of patients with low PTH) demonstrated a decrease in serum calcium levels 24 h after surgery (Table 2). By 48 h after surgery, all 53 patients with low post-operative PTH had low calcium (Table 3, *p* < 0.001). As previously described, patients with a reduction in serum PTH or calcium after surgery were treated with calcium and vitamin D supplementation. We did not report the time-course of the calcium levels of those patients because the calcium values were influenced by the calcium and vitamin D supplementation. In 88 patients (62.4%), the PTH level remained within the normal range (15–65 pg/mL) after thyroidectomy with a mean level of 40.7 ± 15.6 pg/mL, followed by normal serum calcium level at 24 h (mean 9.6 ± 0.6 mg/dl) and at 48 h (mean 8.9 ± 0.4 mg/dl). None of these patients required calcium or vitamin D supplementation. Moreover, all patients presented normal values of serum magnesium before and after thyroidectomy; no magnesium supplementation was required.

### 3.3. Influence of MNG, TC and TG on PTH Levels

As previously described, 13 patients showed normal values of calcium 24 h after thyroidectomy despite reduced levels of PTH, but after 48 h, all patients with a decreased PTH after surgery had low calcium. Our data showed that the positive predictive value (PPV) for PTH was 75.5% at 24 h and 100% at 48 h. To evaluate the role of thyroid pathology (MNG, TC and TG) on PTH levels, we performed a one-way ANOVA test. In Table 3 are reported the serum PTH values detected before and after thyroidectomy with respect to the thyroid disease. The one-way ANOVA test showed us that no statistical difference was detected among the groups. In particular, no difference in PTH values was detected between patients affected by MNG, TC or TG before surgery (*p* = 0.93) nor after surgery (Table 4, *p* = 0.06).

## 4. Discussion

The predominant indications for thyroidectomy are thyroid cancer, symptoms such as compression of the trachea or difficulty swallowing, or thyroid dysfunction involving the production of excess thyroid hormone (e.g., toxic nodule, toxic multinodular goiter, and Graves’ disease). Although thyroidectomy is now considered a very safe surgery, known complications can seriously compromise patients’ quality of life [16]. Postoperative hemorrhage [17,18], recurrent laryngeal nerve injury (RLN) [19] and hypocalcemia [20,21] represent the most common adverse events after thyroidectomy. In this study, we focus on the risk of postoperative hypocalcemia. This condition, in fact, represents the most frequent complication of thyroid surgery with an incidence of up to 50% following total thyroidectomy [22,23,24,25]. Post-thyroidectomy hypocalcemia is typically transient, but rarely can become permanent. It can cause serious symptoms and increases the duration of hospitalization. Thus, its prevention is a key goal in thyroid surgery. As previously described, hypocalcemia is the result of hypoparathyroidism, due to injury, removal, or accidental devascularization of the parathyroid glands during surgery [26]. Previous studies have reported that multiple factors, such as the type of disease, surgical technique, and the surgeon’s experience, may contribute to rates of hypoparathyroidism [27,28]. Locally invasive thyroid cancer requires more extensive surgery and increases the risk of complications such as hypocalcemia [28,29]. In the case of cervical lymph node involvement, neck dissection must be performed in addition to thyroidectomy, which also increases the risk of post-operative hypocalcemia [30,31,32]. In our study, to minimize variability due to type of surgery, we only included patients undergoing total thyroidectomy alone. Moreover, surgeon experience is significantly associated with post-operative hypocalcemia [33]. To avoid the influence of surgeon experience, we included only the patients of two surgeons with comparable endocrine surgery experience. The best time to evaluate PTH after thyroidectomy in order to accurately predict post-operative hypocalcemia remains under debate. The short half-life of PTH is well known, and this property is frequently utilized during parathyroidectomy, wherein serum PTH is typically detected just 10 min after parathyroid removal [34,35]. However, current literature suggests that PTH evaluation 3–4 h after thyroidectomy may be optimal for predicting post-operative hypocalcemia [36,37,38]. In this study, serum PTH drawn 3 h after surgery was predictive of subsequent hypocalcemia. In 26.1% of patients underwent total thyroidectomy, we observed a reduction in PTH 3 h after thyroidectomy, followed by a decrease in serum calcium levels with an average PPV for PTH of 77.8% at 24 h and 100% at 48 h after the thyroidectomy. There was a significant correlation between the 3-h postoperative PTH level and the development of hypocalcemia within 48 h after surgery (*p* < 0.001). We demonstrated that a single measurement of serum PTH performed 3 h after thyroidectomy was able to predict the development of hypocalcemia 48 h in advance with an accuracy of 100%. These data suggest that all patients with low levels of PTH after thyroidectomy could benefit from early supplementation of calcium and vitamin D to prevent the development of clinically significant hypocalcemia. The prevention of hypocalcemia would be expected to reduce readmission for hypocalcemic symptoms, as well as the related hospitalization costs [39]. Although low PTH after surgery has been previously shown to be associated with symptomatic hypocalcemia, the routine analysis of post-thyroidectomy PTH is not universally accepted. The major criticism is the lack of a clear PTH threshold for both calcium and vitamin D supplementation. Some authors suggest that PTH may not be the only determinant for the development of hypocalcemia and that other factors may play a major role such as vitamin D deficiency [40], dietary habits [41], and other metabolic disorders such as hypomagnesemia [42,43]. Hypomagnesemia is frequently associated with the use of drugs such as aminoglycosides, cyclosporine, cisplatin, amphotericin B, ACE inhibitors, proton pump inhibitors, and some diuretics (thiazides and loop) [44]. The metabolism of magnesium is also influenced by conditions causing the alteration of plasma volume, such as the osmotic diuresis observed in diabetes mellitus or increased gastrointestinal losses, as well as in malabsorption syndromes [45]. Consequently, all these factors must be considered in order to prevent post-thyroidectomy hypocalcemia, while PTH evaluation should remain a marker for starting calcium and vitamin D supplementation after surgery. We also showed that the serum PTH predicts post-thyroidectomy hypocalcemia regardless of thyroid disease; no difference was observed in patients affected by MNG, TC or GD. Our data suggest that the detection of PTH 3 h after thyroidectomy is a reliable marker to predict post-surgical hypocalcemia and to prevent overtreatment of normocalcemia that occurs with routine calcium and/or vitamin D supplementation. Moreover, the routine use of PTH for the early identification of hypocalcemia may help reduce the length of hospitalization and associated costs, though further research will be required.

## 5. Conclusions

PTH <15 pg/mL at 3 h after total thyroidectomy accurately predicts post-operative hypocalcemia. The early detection of patients at risk of developing post-operative hypocalcemia allows for prompt supplementation of calcium and Vitamin D in order to prevent symptoms and allows for a safe and timely discharge.

## Figures and Tables

**Table 1:** Patient characteristics.

CATEGORY (*n* = 141)	PATIENTS (N)	PATIENTS (%)
**Gender**		
Male	35	24.8
Female	106	75.2
**Diagnosis**		
MNG	48	34.1
TC	46	32.6
TG	47	33.3
Age (Mean ± SD)	48.73 ± 13.44	

Abbreviations: MNG: multinodular goiter, TC: thyroid cancer, TG: Graves’ disease and toxic (multi)nodular goiter.

**Table 2:** Serum levels of PTH and the calcium after thyroidectomy.

	BEFORE THYROIDECTOMY				AFTER THYROIDECTOMY			
	**PTH**		**CALCIUM**		**PTH 3 HOURS**		**CALCIUM 24 HOURS**	
**SERUM** **LEVELS**	Normal (15–65 pg/mL)	Reduced (<15 pg/mL)	Normal (8.5–10.5 mg/dL)	Reduced (<8.5 mg/dL)	Normal (15–65 pg/mL)	Reduced (<15 pg/mL)	Normal (8.5–10.5 mg/dL)	Reduced (<8.5 mg/dL)
**Patients**	141 (100%)	0 (0%)	141 (100%)	0 (0%)	88 (62.4%)	53 (37.6%)	101 (71.6%)	40 (28.4%)

**Table 3:** Serum calcium levels in patients with low PTH after thyroidectomy.

PTH REDUCED 3 HOURS AFTER THYROIDECTOMY (*n* = 53)				
	***CALCIUM 24 HOURS*** ***after Thyroidectomy***		***CALCIUM 48 HOURS*** ***after Thyroidectomy***	
**SERUM** **LEVELS**	Normal (8.5–10.5 mg/dL)	Reduced (<8.5 mg/dL)	Normal (8.5–10.5 mg/dL)	Reduced (<8.5 mg/dL)
**Patients**	13 (24.5%)	40 (75.5%)	0 (0%)	53 (100%)

**Table 4:** Post-operative serum PTH, by thyroid pathology.

DIAGNOSIS	PATIENTS N	NORMAL PTH VALUES N (%)	REDUCED PTH VALUES N (%)
MNG	48	30 (62.5%)	18 (37.5%)
TC	46	31 (69.6%)	15 (32.6%)
TG	47	27 (77.3%)	20 (42.5%)

## Data Availability

The data presented in this study are available on request from the corresponding author. The data are not publicly available due to privacy.
